# The phosphorylation of a kinetochore protein Dam1 by Aurora B/Ipl1 kinase promotes chromosome bipolar attachment in yeast

**DOI:** 10.1038/s41598-017-12329-z

**Published:** 2017-09-19

**Authors:** Fengzhi Jin, Michael Bokros, Yanchang Wang

**Affiliations:** 1 Department of Biomedical Sciences, College of Medicine, Florida State University, 1115 West Call Street, Tallahassee, FL 32306-4300 USA; 2 Present Address: Yerkes National Primate Research Center, Emory Vaccine Center, 954 Gatewood Rd NE, Atlanta, GA 30329 USA

## Abstract

The interaction between chromosomes and spindle microtubules is essential for chromosome segregation. The kinetochore complex mediates this interaction. Previous studies indicate that the stability of kinetochore attachment is regulated by Aurora B/Ipl1 kinase and this regulation is conserved from yeast to mammalian cells. In budding yeast *Saccharomyces cerevisiae*, the ten-subunit Dam1/DASH complex bridges the interaction between kinetochores and microtubules, and some *in vitro* evidence indicates that the phosphorylation of Dam1 protein by Ipl1 kinase destabilizes this interaction. However, it is not clear if Dam1 phosphorylation is sufficient to regulate the stability of kinetochore attachment *in vivo*. Also, the significance of this regulation in response to chromosome detachment has not been fully investigated. Here we report that phospho-deficient *dam1-3A* mutants show stabilized kinetochore-microtubule attachment *in vivo*. This significantly delays the establishment of chromosome bipolar attachment after the disruption of kinetochore-microtubule interaction by a microtubule depolymerizing drug nocodazole. Moreover, *dam1-3A* cells show dramatic chromosome mis-segregation after treatment with nocodazole, presumably due to the combination of compromised bipolar attachment and premature spindle assembly checkpoint silencing in the mutant cells. Therefore, the regulation of Dam1 phosphorylation imposed by Ipl1 kinase is critical for faithful chromosome segregation.

## Introduction

The kinetochore is a multiple protein complex that mediates the interaction between chromosomes and spindle microtubules. The establishment of chromosome bipolar attachment is essential for the separation of sister chromatids and mistakes in this process result in chromosome mis-segregation, causing aneuploidy, a hallmark for most solid tumors^1^. Different from other kinetochore proteins, yeast Dam1/DASH complex oligomerizes *in vitro* to form a ring that encircles microtubules, thus the interaction of microtubule-associated Dam1 complex with other kinetochore proteins establishes kinetochore-microtubule interaction^2,3^. Recent evidence indicates that the Ndc80 kinetochore protein complex interacts with three proteins in the Dam1 complex to mediate kinetochore-microtubule interaction^4^. In this way, the Dam1 complex bridges the kinetochore and the microtubule. After DNA duplication, sister kinetochores establish bipolar attachment for accurate chromosome segregation. Errors in chromosome attachments must be resolved, but an important question is how cells correct these incorrect attachments.

In budding yeast, compromised function of Ipl1 kinase results in stabilized kinetochore attachment^5^. Since incorrect attachments prevent tension generation on chromosomes, it is speculated that Ipl1 kinase destabilizes tensionless chromosome attachment, which not only facilitates the correction of erroneous attachments, but also activates the spindle assembly checkpoint (SAC) to delay anaphase onset^6^. In mammalian cells, incorrect kinetochore-microtubule interaction is also stabilized when Aurora B kinase (the Ipl1 homologue) is inhibited^7,8^. Therefore, yeast and mammalian cells likely share a similar mechanism to regulate the stability of kinetochore attachment by Aurora B/Ipl1 kinase. This kinase is a component of the conserved chromosome passenger complex (CPC) that is translocated from chromosomes to the spindle during anaphase. In addition to the Aurora B/Ipl1 kinase, the CPC also includes inner centromere protein INCENP/Sli15, and two other regulatory subunits Survivin/Bir1, and Borealin/Nbl1^9,10^. Previous studies indicate that Aurora B/Ipl1 kinase regulates the stability of kinetochore-microtubule interaction by phosphorylating multiple proteins in the Dam1 complex in yeast cells.

The yeast Dam1/DASH complex consists of tensubunits^11–13^. A systemic investigation of the kinetochore proteins that are phosphorylated by Ipl1 kinase identified three subunits of the Dam1 complex, including Dam1, Ask1, and Spc34 ﻿proteins. Among these three proteins, mutations of the Ipl1 consensus sites in Dam1, but not in Ask1 or Spc34, result in lethality^14^. Although replacement of all four Ipl1 consensus sites (S^20^ S^257^ S^265^ S^292^) in Dam1 protein with Ala (A) or Asp (D) causes lethality, mutations in three of the four sites (S^257^ S^265^ S^292^) generates viable phospho-deficient (*dam1-3A*) and phospho-mimetic (*dam1-3D*) mutants. The *dam1-3A* mutants show normal growth, but are sensitive to microtubule depolymerizing agent benomyl, whereas *dam1-3D* mutants exhibit sick growth phenotype. Moreover, *dam1* (S20A S292A) and *dam1* (S257A S265A S292A) showed synthetic temperature sensitivity in combination with *spc34* (T199A). In many cases, more than 90% of the DNA was segregated to a single pole during anaphase in these double phospho-mutants. In addition, the phospho-mimetic *dam1* mutations suppress the temperature sensitivity of *ipl1-2*, indicating that Dam1 is a critical substrate of Ipl1 kinase that regulates chromosome segregation^14^. Using bacterially expressed proteins, a previous study showed that phospho-mimetic mutation of the Ipl1 consensus sites in Dam1 (S to D) reduces its binding to Ndc80 kinetochore complex^15^. Moreover, the Dam1 complex recruits the Ndc80 complex to the microtubule *in vitro*, but the phosphorylation of the Dam1 complex by Ipl1 kinase abolishes this recruitment, indicating the negative role of Dam1 phosphorylation by Ipl1 in kinetochore-microtubule attachment^16,17^.

In addition to the regulation of the stability of kinetochore attachment, our recent evidence indicates that Dam1 protein phosphorylation also regulates the activity of the spindle assembly checkpoint (SAC). In the absence of sister chromatid cohesion, cells fail to generate tension on chromosomes. The same is true when sister kinetochores are attached by microtubules from the same spindle pole (syntelic attachment). Dysfunctional Ipl1 kinase abolishes the anaphase entry delay caused by the lack of cohesion^18^. We found that phospho-deficient *dam1-3A* mutant also abolishes the anaphase entry delay induced by cohesion defect or syntelic attachment^19,20^, indicating that Dam1 phosphorylation upregulates SAC activity. Here, we present *in vivo* evidence showing stabilized kinetochore attachment in *dam1-3A* mutant cells. Moreover, *dam1-3A* cells exhibit much slower kinetics in the establishment of chromosome bipolar attachment after exposure to spindle poisons. In addition, *dam1-3A* mutant cells lose viability and show chromosome mis-segregation after exposure to spindle poisons. These observations support the conclusion that Dam1 phosphorylation not only destabilizes kinetochore attachment to facilitate chromosome bipolar attachment, but also prevents anaphase onset in the presence of tensionless attachment. Therefore, timely regulated phosphorylation of Dam1 protein is critical for faithful chromosome segregation.

## Results

### Phospho-deficient *dam1* mutant cells lose viability after exposure to spindle poison

**Figure 1:**
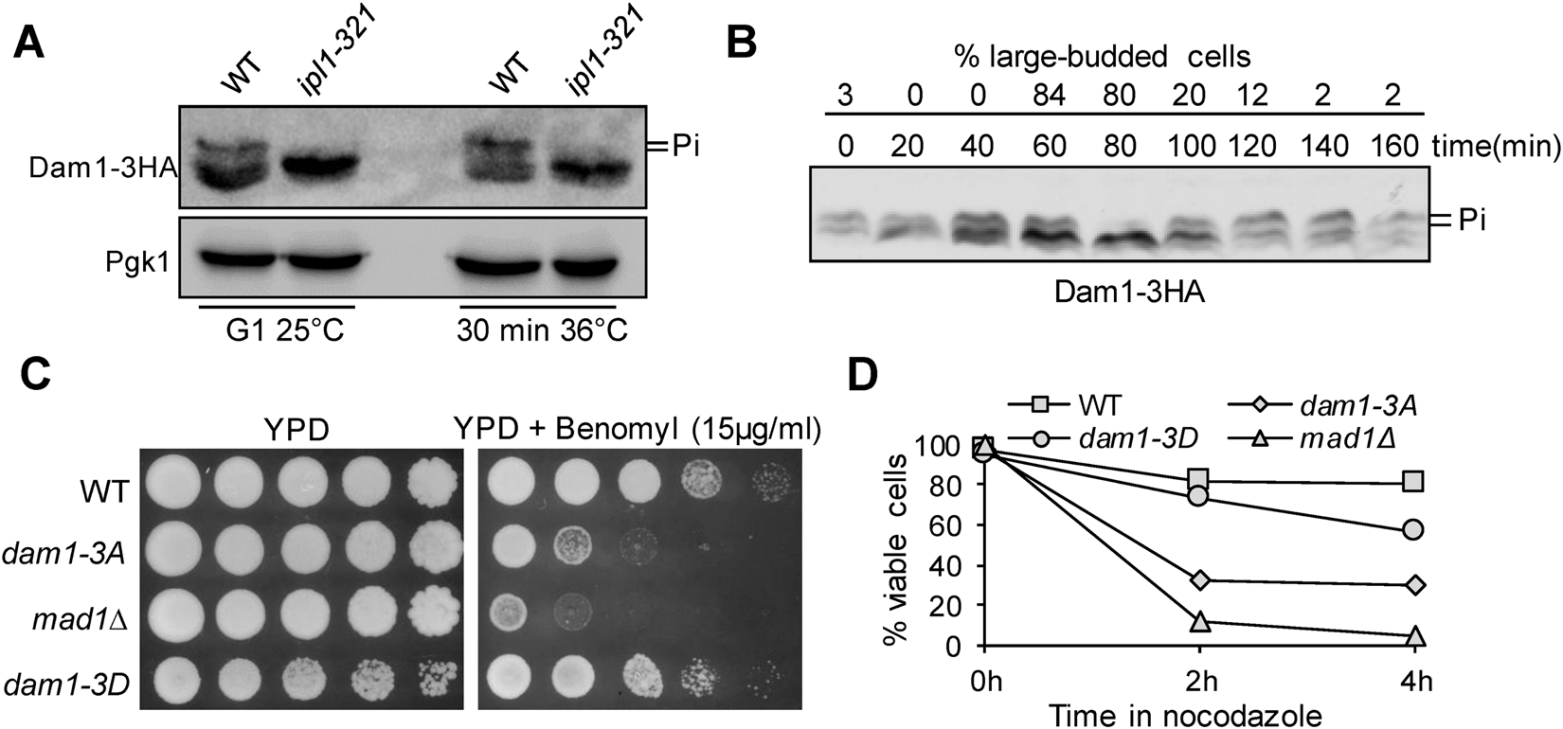
The phosphorylation of Dam1 is Ipl1-dependent and cell cycle regulated. (**A**) Dam1 phosphorylation depends on Ipl1 kinase. *DAM1-3HA* and *ipl1*-*321 DAM1-3HA* cells were arrested in G_1_ phase with α-factor at 25 °C, then released into to 36 °C YPD (yeast extract peptone dextrose). Protein samples were prepared at the indicated time points for western blotting with anti-HA and anti-Pgk1 antibodies. Cropped gels are displayed. The same samples were used for anti-HA and anti-Pgk1 blots but were from two separate gels. (**B**) Dam1 dephosphorylation during the cell cycle. *DAM1-3HA* cells were synchronized in G_1_ and then released into 30 °C YPD medium. α-factor was added back after budding. The cells were collected over time to examine Dam1 phosphorylation based on the band-shift after western blotting. The percentage of large budded cells is shown on the top. A cropped gel is displayed. (**C**) The phospho-deficient mutant *dam1-3A* is sensitive to benomyl. Saturated WT and mutant cells were 10-fold serial diluted, spotted onto YPD plates with or without Benomyl (15 µg/ml) and incubated at 30 °C for 2 days. (**D**) *dam1-3A* mutant cells lose viability after nocodazole treatment. Log-phase cells with the indicated genotypes were treated with 20 µg/ml nocodazole at 30 °C. At time 0, 2 and 4 hr, samples were collected and spread onto YPD plates to examine the plating efficiency after overnight growth at room temperature (n ≥ 200).

Kinetochore protein Dam1 is one of the subunits in the Dam1/DASH complex and it is involved in both kinetochore-microtubule interaction and SAC checkpoint regulation^21^. Previous data indicate that Dam1 is phosphorylated by Ipl1 kinase^11,14,22^, but the timing of Dam1 dephosphorylation remains unclear. To clarify this, we generated *DAM1-3HA* strains using a PCR protocol^23^. The tagged Dam1 proteins showed a clear band-shift, but the slow-migrating bands were not detectable in *ipl1-*321 mutant cells arrested in G_1_ phase at 25 °C. After release into cell cycle at 36 °C for 30 min, the slow-migrating bands were persistent in wild-type (WT) cells, but these bands were not visible in *ipl1-321* mutant cells, indicating that the band-shift depends on Ipl1 kinase (Fig. 1A). We then examined Dam1 phosphorylation in synchronized WT cells incubated at 30 °C. After G_1_ release for 80 min, most of the slow-migrating bands disappeared abruptly and appeared again at 100 min. Therefore, Dam1 protein is dephosphorylated within a narrow window during the cell cycle (Fig. 1B). It is likely that the M to G1 transition occurs between 80 and 100 min because of the decrease of the population of large-budded cells. Thus, we speculate that Dam1 dephosphorylation occurs during anaphase, and our previous observation that the protein level of anaphase inhibitor Pds1 decreases during this window supports this speculation^24^.

To understand the function of Ipl1-dependent Dam1 phosphorylation, we examined the sensitivity of phospho-deficient *dam1-3A* mutant cells to spindle poison. *dam1-3A* cells grew as well as WT cells on YPD (Yeast extract peptone dextrose) plates, but *dam1-3A* mutants exhibited sick growth on YPD plates containing benomyl, a microtubule depolymerizing drug. As a control, SAC mutant *mad1*∆ also exhibited sensitivity to benomyl. In contrast, the phospho-mimetic mutant *dam1-3D* grew as well as WT cells on ﻿benomyl plates(Fig. 1C). Consistently, both *dam1-3A* and *mad1*∆, but not *dam1-3D*, showed significant viability loss after exposure to nocodazole, another microtubule depolymerizing agent (Fig. 1D). After 4 hr treatment with 20 µg/ml nocodazole, 70% of *dam1-3A* cells and 95% *mad1∆* cells lost viability, indicating that Ipl1-dependent Dam1 phosphorylation is required for yeast cell survival following disruption of the kinetochore-microtubule interaction.

### Dephosphorylation of Dam1 stabilizes kinetochore-microtubule attachment

The sensitivity of *dam1-3A* mutants to spindle poisons could be a result of defective kinetochore attachment or checkpoint failure. Previous data show that *ipl1* mutants exhibit stabilized kinetochore attachment^5^, and this phenotype is likely due to compromised phosphorylation of Dam1 and/or other kinetochore proteins^11,14^. Therefore, phospho-deficient *dam1-3A* mutants may behave similarly as *ipl1* mutants and show stabilized kinetochore attachment. To test this possibility *in vivo*, we examined chromosome segregation in cells with *P*_*GAL*_ *HA-CDC6* (*CDC6* gene under a galactose-inducible promotor control) incubated in glucose medium, which represses *CDC6* gene transcription and blocks DNA synthesis as Cdc6 protein is essential for the initiation of DNA replication^25^. Because the unduplicated chromosomes connect to the “old” spindle pole that enters daughter cells^26^, only the turnover of kinetochore-microtubule interaction allows random segregation of unduplicated chromosomes into both mother and daughter cells. Therefore, the examination of the distribution of unduplicated chromosomes in mother and daughter cells could determine the stability of chromosome attachment^5^.

**Figure 2:**
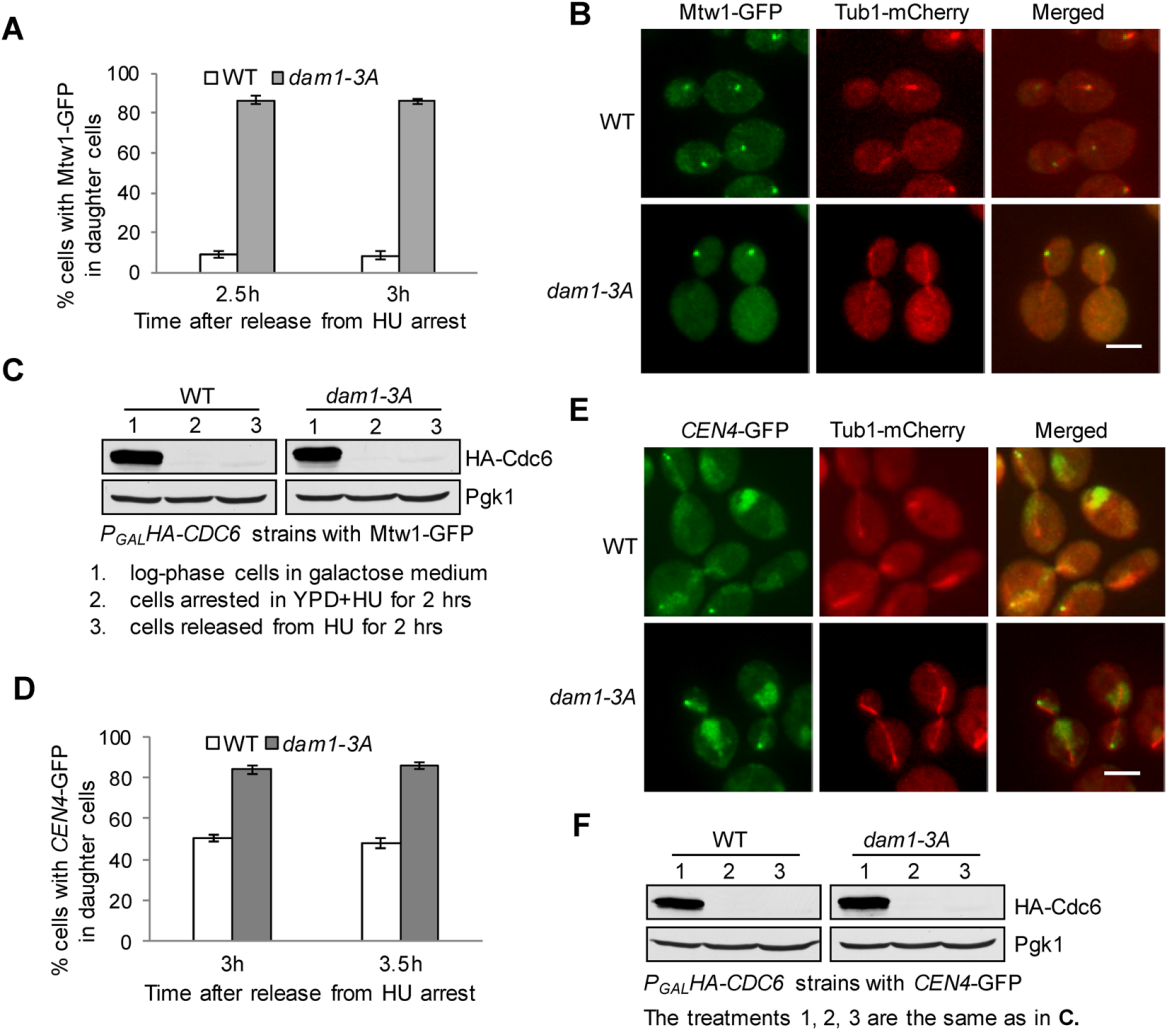
Dam1 dephosphorylation stabilizes kinetochore attachment. **(A)** WT and *dam1-3A* cells with *P*_*GAL*_ *HA-CDC6 MTW1*-*GFP* *TUB1*-*mCherry* were grown in YEP-GAL (Yeast extract peptone galactose) to log-phase at 30 °C, then released into YPD medium containing 200 mM hydroxyurea (HU) for 2 hr. after HU was washed off, the cells were released into YPD. Time 0 is when HU was washed off. Cells were collected at the indicated time points after HU wash-off and fixed for the examination of fluorescent signals. The percentage of cells with Mtw1-GFP signals only in daughter cells was counted (n ≥ 100). **(B)** Representative images show the spindle morphology and Mtw1-GFP distribution in WT and *dam1-3A* cells (from A). Daughter cells are usually smaller than mother cells. **(C)** The degradation of Cdc6 protein. The cells were treated as described above and the protein samples were prepared at the indicated time points to determine HA-Cdc6 protein levels using western blotting. Pgk1 protein levels were used as a loading control. Cropped gels are displayed. **(D)** WT and *dam1-3A* cells with *P*_*GAL*_ *HA-CDC6 CEN4*-*GFP* *TUB1*-*mCherry* were grown in galactose medium to log-phase. The cells were then released into YPD containing 200 mM HU for 2 hrs at 30 °C. After HU wash-off, the cells were collected at 3 and 3.5 hrs for the examination of fluorescent signals. The percentage of cells with *CEN4*-GFP localized in daughter cells was counted (n ≥ 100). **(E)** The spindle morphology and the localization of *CEN4*-GFP in some representative cells. **(F)** The degradation of Cdc6. The cells were collected at the indicated time points to determine the protein level of Cdc6 and Pgk1. Cropped gels are displayed.

WT and *dam1-3A* mutant cells with *P*_*GAL*_ *HA-CDC6* were grown in galactose medium and then switched into glucose medium (YPD) containing 200 mM DNA synthesis inhibitor hydroxyurea (HU) for 2 hrs, which represses *CDC6* expression and synchronizes cells in S-phase. The cells were then released into YPD medium without HU and allowed to finish their first mitosis. We monitored chromosome segregation in the following cell cycle by examining the localization of GFP-tagged kinetochore protein Mtw1^27,28^. The reason we examined chromosome segregation in the following cell cycle is to ensure a complete Cdc6 depletion before the initiation S-phase in the succeeding cell cycle. We found that most of the WT cells showed two GFP clusters in mother and daughter cells, indicating random chromosome segregation. In clear contrast, 86% of *dam1-3A* cells showed a single GFP cluster in the daughter cell (Fig. 2A and B), indicating stabilized association of unduplicated chromosomes with the “old” spindle pole. Here, we distinguished the mother and daughter cells based on their size, as daughter cells are usually smaller. To confirm that Cdc6 protein was degraded efficiently after release into glucose medium, we examined Cdc6 protein level in the cells at different experimental stages. We found that Cdc6 protein disappeared when cells were arrested in glucose medium containing HU for 2 hrs (Fig. 2C). Therefore, Cdc6 was depleted when cells initiated the second round of cell cycle, which ensures the complete block of DNA replication and the formation of sister kinetochores.

To further confirm the stable kinetochore attachment in *dam1-3A* cells, we also examined the segregation of unduplicated chromosomes using strains with GFP-marked centromere of chromosome IV (*CEN4*-GFP)^29^. WT and *dam1-3A* mutant cells with *P*_*GAL*_ *HA-CDC6 CEN4-GFP TUB1-mCherry* were treated as described above. After release from HU arrest for 3 hrs, 50% of WT cells showed daughter cell localization of *CEN4*-GFP, but 84% of *dam1-3A* cells showed *CEN4*-GFP in the daughter cell. We also collected cells after HU release for 3.5 hrs and the result was similar (Fig. 2D and E), suggesting that the majority of the unduplicated chromosomes in *dam1-3A* mutant cells maintain their association with the “old” spindle pole. In these strains, Cdc6 protein degradation was efficient when cells were shifted from galactose medium to YPD (Fig. 2F). Therefore, our data support the conclusion that abolishment of Ipl1-dependent Dam1 phosphorylation is sufficient to stabilize kinetochore-microtubule interaction *in vivo*.

### Dam1 phosphorylation is required for efficient chromosome biorientation

**Figure 3:**
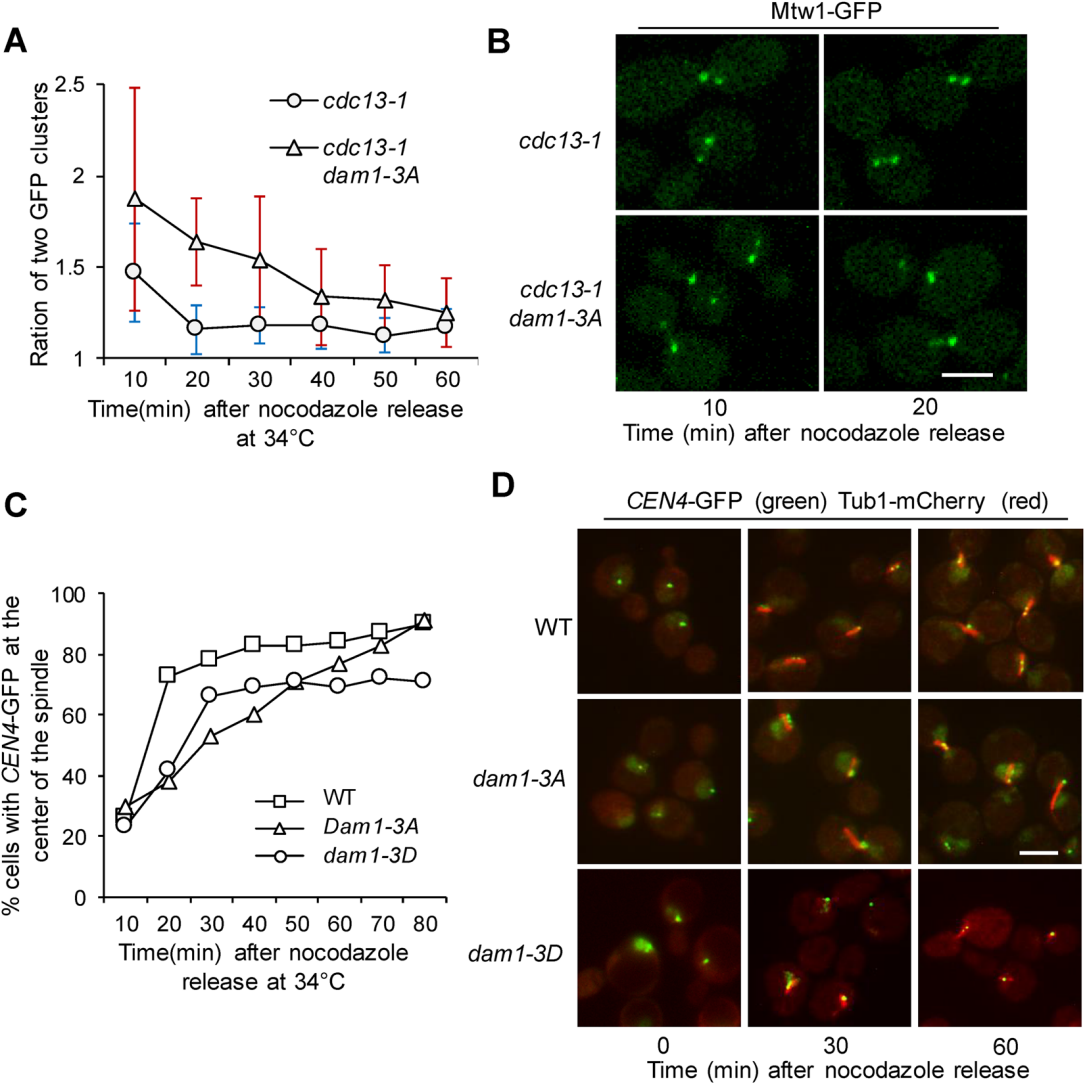
Dam1 phosphorylation is required for the establishment of chromosome bipolar attachment after nocodazole treatment. **(A)** G_1_-arrested *cdc13-1* and *cdc13-1 dam1-3A* cells with *MTW1-GFP TUB1-mCherry* were released into YPD medium containing 20 µg/ml nocodazole at 34 °C for 2hr. Nocodazole was washed off, and then cells were released into YPD at 34 °C. Cells were collected every 10 min and fixed for the examination of fluorescent signals. Confocal microscopy was used for the projection of maximum-intensity images. The Mtw1-GFP fluorescence density of each kinetochore cluster was determined, and the average ratio of the intensity between the two kinetochore clusters in each cell was shown (n > 20). **(B)** Representative images for the Mtw1-GFP signal acquired by confocal microscopy after nocodazole was washed off for 10 and 20 min. Scale bar: 5 µm. **(C)** *cdc13-1, cdc13-1 dam1-3A* and *cdc13-1 dam1-3D* cells with *CEN4*-*GFP TUB1*-*mCherry* were treated as described in A. The percentage of cells with *CEN4*-GFP localized at the center region of the spindle is shown. **(D)** The relative localization of *CEN4*-GFP (green) to the spindle (red) in some representative cells are shown. Scale bar: 5 µm.

We found that *dam1-3A* cells exhibited viability loss after treatment with nocodazole (Fig. 1). It is possible that the stabilized kinetochore attachment impairs the establishment of chromosome bipolar attachment due to the failure to correct erroneous attachment induced by nocodazole treatment. To investigate the chromosome biorientation in *dam1-3A* mutants, we used cells arrested in pre-anaphase by *cdc13-1*. Cdc13 binds to telomeres and protects chromosome ends^30^. In *cdc13-1* mutants incubated at non-permissive temperatures, unprotected telomeres activate the DNA damage checkpoint to arrest cells in pre-anaphase with established chromosome bipolar attachment^31,32^. To follow the process of chromosome bipolar attachment, WT and *dam1-3A* mutant cells with Mtw1-GFP and Tub1-mCherry were arrested in G_1_ phase and then released into 34 °C medium containing nocodazole for 2 hr. The majority of cells were arrested as large budded cells. Nocodazole was then washed off and the cells were maintained at 34 °C. In yeast cells, the establishment of chromosome bipolar attachment leads to the formation of two kinetochore clusters, and the intensity of the two clusters should be similar^33^. Using a confocal microscope, we measured the intensity ratio of the two kinetochore clusters in each cell over time after nocodazole wash-off. More than 20 cells were examined for each time point. We did not count the ratio at time 0, because no separated kinetochore clusters were present due to the lack of spindle structure. After nocodazole wash-off for 10 min, the average ratio for WT cells was 1.47, and it was 1.87 for *dam1-3A* mutant cells (Fig. 3A,B), indicating more unequal distribution of kinetochores in the two clusters in *dam1-3A* mutant cells. Although the ratio was close to 1 in both WT and *dam1-3A* cells after nocodazole wash-off for 60 min, it was clear that WT cells take much less time to reach this point. Fluorescence microscopy confirmed that no spindle structure was visible just after nocodazole release (Time 0), but the spindle structure appeared after 10 min release from nocodazole treatment. Therefore, the results suggest that the establishment of chromosome bipolar attachment is less efficient in *dam1-3A* cells after nocodazole exposure.

To further determine the role of Dam1 phosphorylation in the establishment of chromosome bipolar attachment, we used *TUB1-mCherry CEN4-GFP* strains to analyze the relative localization of *CEN4*-GFP to the spindle after exposure to nocodazole. Before bipolar attachment, the *CEN4*-GFP signal usually colocalizes with one end of the spindle. After bipolar attachment, the *CEN4*-GFP signal moves to the center region of the spindle, and two GFP dots can be observed in some cells due to the tension on sister centromeres^29^. Therefore, G_1_-synchronized *cdc13-1, cdc13-1 dam1-3A* and *cdc13-1 dam1-3D* cells with *TUB1-mCherry CEN4-GFP* were released into 34 °C medium containing nocodazole for 2 hrs. The cells were then switched to 34 °C medium without nocodazole and collected to visualize t he relative *CEN4*-GFP localization to spindle. After 10 min release, less than 30% of cells showed *CEN4*-GFP localization at the center region of the spindle. However, this number increased to 73% for WT cells, but only 38% to *dam1-3A* cells after nocodazole release for 20 min. Compared to WT cells, *dam1-3A* cells showed a slower rate to achieve *CEN4*-GFP localization at spindle center region (Fig. 3C and D). Just after nocodazole wash-off, the establishment of chromosome bipolar attachment in *dam1-3D* cells appeared to be less efficient than WT cells, but more efficient than *dam1-3A* cells. At later time points of this experiment, however, a larger portion of *dam1-3D* cells were still unable to establish bipolar attachment (Fig. 3D). We reason that the unstable kinetochore attachment in *dam1-3D* cells contributes to this phenotype. Together, these observations support the conclusion that phospho-deficient *dam1-3A* cells have difficulty establishing chromosome bipolar attachment.

### Phospho-deficient *dam1-3A* mutants show chromosome mis-segregation after nocodazole exposure

It appears that *dam1-3A* cells exhibit slow kinetics for the establishment of chromosome bipolar attachment after nocodazole exposure, presumably due to stabilized kinetochore attachment. We previously showed that *dam1-3A* cells silence the SAC prematurely in the presence of tensionless kinetochore attachment^20^, which could also contribute to the viability loss in *dam1-3A* cells treated with nocodazole in addition to the impaired chromosome bipolar attachment. We first compared the cell cycle progression in WT and *dam1-3A* cells after exposure to nocodazole. Surprisingly, *dam1-3A* mutant cells exhibited slightly delayed degradation of anaphase inhibitor Pds1 than WT cells. Thus, no premature anaphase entry was detected by examining the Pds1 level in a population of *dam1-3A* cells after nocodazole treatment. It is possible that measuring Pds1 degradation is not sensitive enough to detect a small population of *dam1-3A* cells that prematurely enter anaphase.

**Figure 4:**
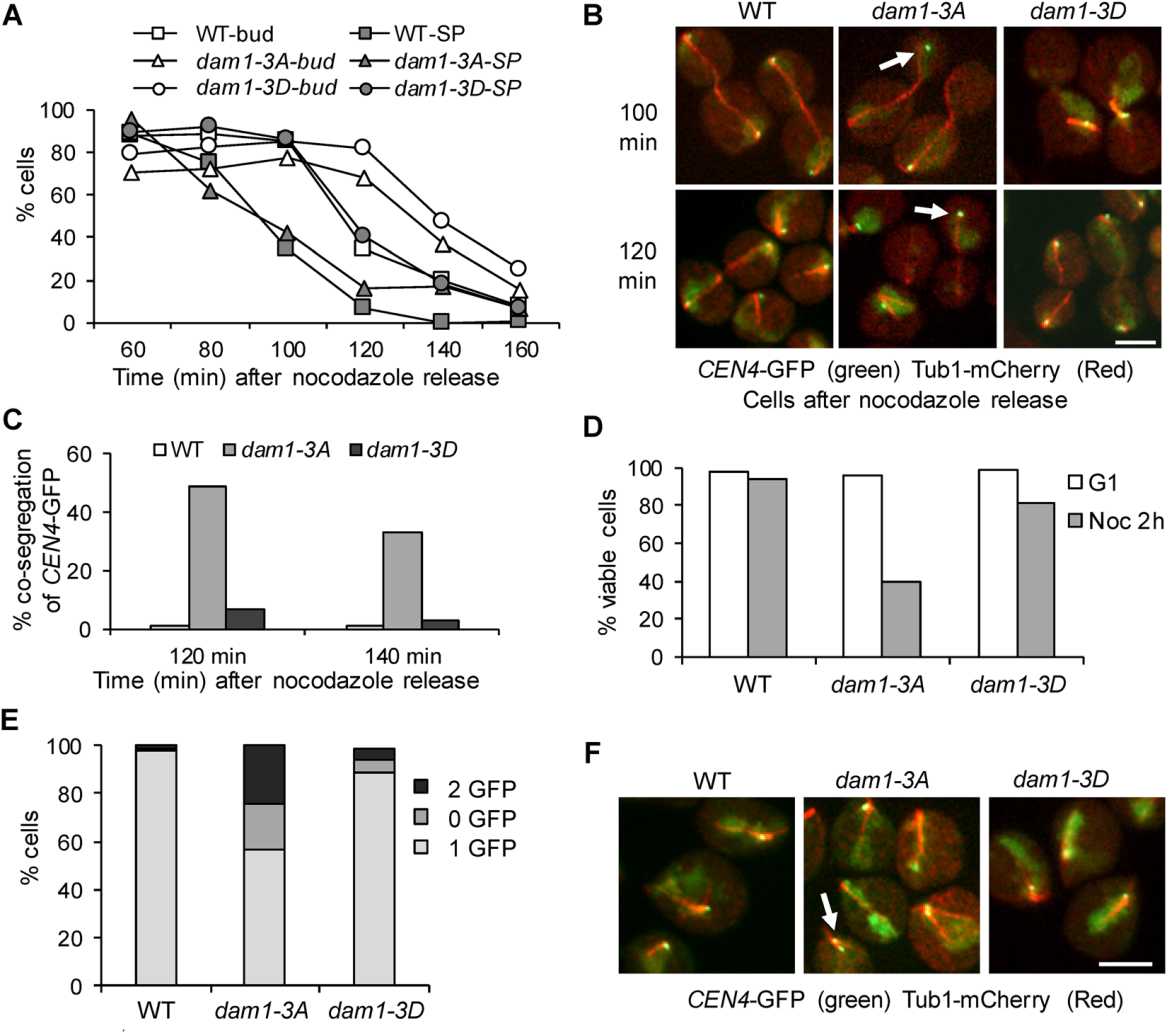
Phospho-deficient *dam1-3A* mutants show chromosome mis-segregation after nocodazole exposure. G_1_-arrested WT, *dam1-3A* and *dam1-3D* cells with *CEN4*-*GFP* *TUB1*-*mCherry* were released into YPD containing 20 µg/ml nocodazole at 30 °C for 2hr. Then, nocodazole was washed off and the cells were released into YPD at 30 °C (Time 0). α-factor was restored to block next cell cycle. Cells were collected every 20 min for the examination of budding index and fluorescent signals. **(A)** The percentage of large-budded cells and cells with a short spindle (SP) after nocodazole release. **(B)** The distribution of *CEN4*–GFP and spindle morphology in representative cells. The arrows indicate cells with co-segregated *CEN4*–GFP. **(C)** The percentage of cells with an elongated spindle as well as co-segregated *CEN4*–GFP at 120 and 140 min (n ≥ 100). **(D)** The viability loss of WT, *dam1-3A* and *dam1-3D* mutants after nocodazole treatment. The cells arrested in G_1_ and cells after nocodazole (Noc) exposure for 2 hrs were spread onto YPD plates to count the plating efficiency after overnight growth at 25 °C (n ≥ 200). **(E)** The number of *CEN4*-GFP dots in G_1_ cells after nocodazole exposure. After nocodazole exposure, the cells were released and allowed to enter next G_1_ phase. The percentage of G_1_ cells with 0, 1, or 2 *CEN4*-GFP dots was counted (n > 100). **(F)**. Representative images show the *CEN4*-GFP signals in WT, *dam1-3A* and *dam1-3D* cells in G_1_ phase. The arrow indicates a G_1_ cell with 2 *CEN4*-GFP dots.

To further understand the cause of the nocodazole sensitivity of *dam1-3A* cells, we examined the chromosome segregation in WT, *dam1-3A* and *dam1-3D* cells after exposure to nocodazole. After nocodazole release for 40 min, the majority of WT and *dam1* mutant cells were large-budded with a short spindle structure. The kinetics for the disappearance of large budded cells was relatively slower in *dam1-3A* and *dam1-3D* cells compared to WT cells. The disappearance of cells with a short spindle in *dam1-3D* mutants was also delayed. In contrast, *dam1-3A* mutants exhibited similar kinetics for the disappearance of cells with a short spindle, but a small portion still showed a short spindle structure at later time points (Fig. 4A). Strikingly, after nocodazole release for 100 min, a significant portion of *dam1-3A* cells with an elongated spindle showed co-segregated *CEN4*-GFP. The rate of co-segregation in *dam1-3A* was 49% and 33% at 120 and 140 min, respectively, but *CEN4*-GFP co-segregation was negligible in WT and *dam1-3D* cells (Fig. 4B and C). Consistently, *dam1-3A* cells showed significant viability loss after exposure to nocodazole (Fig. 4D). We further allowed the cells to finish anaphase and enter the following G_1_ phase, and then examined the number of *CEN4*-GFP dots in G_1_ cells. Most of the WT and *dam1-3D* cells showed a single GFP dot, indicating even chromosome segregation. In *dam1-3A* cells, however, 19% showed no GFP dot, and 24% exhibited 2 GFP dots (Fig. 4E and F), which confirms frequent chromosome mis-segregation in *dam1-3A* cells following exposure to nocodazole. Together, these results suggest that *dam1-3A* cells have difficulty in establishing chromosome bipolar attachment due to stabilized kinetochore attachment. Moreover, *dam1-3A* mutation likely allows cells with syntelic attachment to enter anaphase, resulting in chromosome mis-segregation.

## Discussion

Previous *in vitro* studies indicate that Ipl1 kinase phosphorylates Dam1 and other kinetochore proteins to destabilize chromosome attachment, but the *in vivo* evidence is less solid and the function of this regulation is still not fully understood. Here we show that phospho-deficient *dam1-3A* mutant cells exhibit stabilized kinetochore attachment using an established protocol from the Nasmyth lab^5^. We found that *dam1-3A* cells showed significantly increased daughter cell localization of the kinetochore cluster (Mtw1-GFP) when DNA synthesis was blocked, indicating a stable association of chromosomes with the “old” spindle pole that primarily moves into daughter cells. Also, the GFP-marked chromosome IV showed higher frequency of daughter-cell localization in *dam1-3A* cells when DNA synthesis is blocked. Therefore, the abolishment of Dam1 phosphorylation in *dam1-3A* seems sufficient to stabilize kinetochore attachment. Moreover, we found that *dam1-3A* cells exhibited slower kinetics for the establishment of chromosome bipolar attachment after treatment with nocodazole. Our explanation is that nocodazole treatment enhances the frequency of erroneous attachment, but stabilized kinetochore attachment in *dam1-3A* cells impairs error correction. Moreover, we found high frequency of chromosome mis-segregation in *dam1-3A* cells after nocodazole treatment, indicating high rate of syntelic attachment and failure of checkpoint arrest. This speculation is supported by our previous observation that *dam1-3A* mutation allows cells with syntelic attachment to enter anaphase due to premature SAC silencing^20,21^.

We suspect that the reversal of Ipl1-dependent Dam1 phosphorylation not only stabilizes kinetochore attachment, but also facilitates SAC inactivation/silencing even in the absence of tension. Compared to the Ipl1-dependent Dam1 phosphorylation, we know little about the Dam1 dephosphorylation. Using synchronized cells, we found an abrupt Dam1 dephosphorylation during the cell cycle, indicating a sudden phosphatase activation or kinase inactivation. It is likely that protein phosphatase 1 (PP1) reverses Ipl1-mediated phosphorylation^34^. PP1 associates with the kinetochore through two proteins, Spc105 and Fin1. The association of PP1 with Spc105 is essential for the SAC silencing, and mutation of the PP1 binding site in Spc105 protein results in lethality due to the failure of SAC silencing^35^. Although Fin1 is also responsible kinetochore recruitment of PP1, this function is not essential for SAC silencing as *fin1*∆ deletion mutants are viable^32,36,37^. It will be our future interest to investigate the role of Spc105- or Fin1-associated PP1 in Dam1 dephosphorylation. Since *dam1-3A* cells show stabilized kinetochore attachment, *dam1-3D* cells are expected to have unstable or weak attachment, but most of *dam1-3D* cells show successful chromosome segregation during anaphase^20^. It is possible that the weak kinetochore attachment in *dam1-3D* cells is sufficient for chromosome segregation, but may cause some problems in the presence of unsolved sister chromatids, which needs to be tested in the future.

Among the ten subunits of the Dam1/DASH complex, Ask1, Spc34 and Dam1 are shown to be Ipl1 substrates. Mutation of the Ipl1 consensus sites in Ask1 does not result in any noticeable phenotypes. Substitution of the Ipl1 consensus sites in Spc34 leads to benomyl sensitivity^14^. In addition to Dam1, it is likely that the phosphorylation of Spc34 also regulates the stability of kinetochore attachment and/or SAC activity. Recent evidence indicates that the phosphorylation of these three proteins by Ipl1 kinase compromises the interaction of the Ndc80 kinetochore complex with two Dam1 complex rings^4,38^. Therefore, it will be interesting to examine the stability of kinetochore attachment and the SAC activity in phospho-deficient *spc34* mutants.

In mammalian cells, the functional orthologue of the Dam1 complex is the Ska (spindle and kinetochore associated), which consists of three subunits (ska1–3) and mediates the interaction between kinetochores and microtubules^39,40^. Aurora B directly phosphorylates ska1 and 3 to impair ska kinetochore recruitment^41^. Therefore, the phosphorylation of ska complex by Aurora B appears to destabilize kinetochore attachment like in yeast cells. However, the ska complex may regulate the SAC activity by promoting the recruitment of anaphase-promoting complex/cyclosome (APC/C) or PP1 to kinetochores^42,43^. In summary, we present *in vivo* evidence showing that the reversal of Ipl1-imposed phosphorylation on Dam1 stabilizes kinetochore-microtubule interaction, and this modification is crucial for the efficient establishment of chromosome bipolar attachment as well as the maintenance of SAC activity, especially after exposure to spindle poisons.

## Materials and Methods

### Yeast strain and growth conditions

The relevant genotypes and the sources of the strains used in this study are listed in Table 1. All of the strains listed are isogenic to Y300, a derivative of W303. The *DAM1-3HA* and *P*_*GAL*_ *HA-CDC6* strains were constructed by using a PCR-based method^23^. For nocodazole treatment, G_1_-arrested cells were release into YPD containing 20 µg/ml nocodazole and 1% DMSO.

**Table 1:** Strain list for this study.

Strains	Relevant genotypes	Reference
Y300	*MAT*a *ura3-1 his3-11,15 leu2-3,112 trp1-1 ade2-1 can1-100*	Lab stock
2376-9-4	*MAT*a *dam1(S257A S265A S292A)::KanMX*	This study
2770-3-4	*MAT*a *dam1(S257D S265D S292D):KanMX*	This study
YYW187	*MAT*a *mad1::HIS3*	Lab stock
2902-3-2	*MAT*a *DAM1-3HA-Sphis5+*	This study
916-1-4	*MAT*a *ipl1-321 DAM1-3HA-Sphis5+*	This study
1091-5-3	*MAT*a *cdc13-1 promURA3::tetR::GFP-LEU2 CENIV::tetOX448::URA3 TUB1-mCherry::URA3*	Lab stock
2442-1-3	*MAT*a *cdc13-1 dam1(S257A S265A S292A)::KanMX promURA3::tetR::GFP-LEU2 CENIV::tetOX448::URA3 TUB1-mCherry::URA3*	This study
2419-3-1	*MAT*a *cdc13-1 dam1(S257D S265D S292D)::KanMX promURA3::tetR::GFP-LEU2 CENIV::tetOX448::URA3 TUB1-mCherry::URA3*	This study
1092-9-2	*MAT*a *cdc13-1 MTW1-3GFP-HIS3 TUB1-mCherry::URA3*	This study
2493-1-1	*MAT*a *cdc13-1 dam1(S257A S265A S292A)::KanMX MTW1-3GFP-HIS3 TUB1-mCherry::URA3*	This study
2962-1-3	*MAT*a *TRP1-P*_*GAL*_ *HA-CDC6 promURA3::tetR::GFP-LEU2 CENIV::tetOX448::URA3 TUB1-mCherry::URA3*	This study
2955-3-2	*MAT*a *TRP1-P*_*GAL*_ *HA-CDC6 dam1(S257A S265A S292A)::KanMX promURA3::tetR::GFP-LEU2 CENIV::tetOX448::URA3 TUB1-mCherry::URA3*	This study
3453-4-1	*MAT*a *TRP1-P*_*GAL*_ *HA-CDC6 MTW1-3GFP-HIS3 TUB1-mCherry::URA3*	This study
3453-5-2	*MAT*a *TRP1-P*_*GAL*_ *HA-CDC6 dam1(S257A S265A S292A)::KanMX MTW1-3GFP-HIS3 TUB1-mCherry::URA3*	This study
YYW141	*MAT*a *promURA3::tetR::GFP-LEU2 CENIV::tetOX448::URA3 TUB1-mCherry::URA3*	Lab stock
2320-2-4	*MAT*a *dam1(S257A S265A S292A)::KanMX promURA3::tetR::GFP-LEU2 CENIV::tetOX448::URA3 TUB1-mCherry::URA3*	This study
2377-1-1	*MAT*a *dam1(S257D S265D S292D)::KanMX promURA3::tetR::GFP-LEU2 CENIV::tetOX448::URA3 TUB1-mCherry::URA3*	This study

### Western blot analysis

We collected 1.5 ml yeast cell culture and the cell pellets were resuspended in 100 µl H_2_O and then 100 µl 0.2 M NaOH was added. The mixture was left at room temperature for 5 min. The pellet was resuspended in the loading buffer. We used 10% Acrylamide gels for SDS-PAGE. The anti-HA (16B12) (Covance Research Products, Inc.) was used at a 1:750 dilution. The anti-Pgk1 antibody (Molecular Probes, Eugene, OR) was used at 1:10,000 dilution. Proteins were detected with ECL (Perkin-Elmar LAS, Inc.).

### Fluorescent signal analysis

Strains containing GFP-labeled centromere of chromosome IV (*CEN4-GFP)* and *TUB1*-*mCherry* were collected and fixed with 3.7% formaldehyde at room temperature for 5 min. The cells were then washed with water and resuspended in 1 × PBS. The fluorescence signal was analyzed in cells with an elongated spindle using a fluorescence microscope (EVOS from Life Technologies). For the ratio of the two Mtw1-GFP clusters in each cell, fixed cells were subjected to confocal microscopy (LAS TCS MP5 from Leica Microsystems). The images are a projection of 10 sections (0.2 µm intervals). The quantification of the GFP signal clusters was analyzed using the LAS AF Lite software.

### Data availability

All data generated or analyzed during this study are included in this published article.

## Electronic supplementary material

Supplementary Information
