# Supplementary material for: The phosphorylation of a kinetochore protein Dam1 by Aurora B/Ipl1 kinase promotes chromosome bipolar attachment in yeast
Source: Sci Rep. 2017 Sep 19;7:11880. doi: 10.1038/s41598-017-12329-z (PMC5605499; doi:10.1038/s41598-017-12329-z)
Supplement: Supplementary file 1 — Supplementary Information [file 41598_2017_12329_MOESM1_ESM.pdf]

Supplementary Information

The phosphorylation of a kinetochore protein Dam1 by Aurora B/Ipl1 kinase promotes chromosome bipolar attachment in yeast

Fengzhi Jin[#], Michael Bokros and Yanchang Wang*

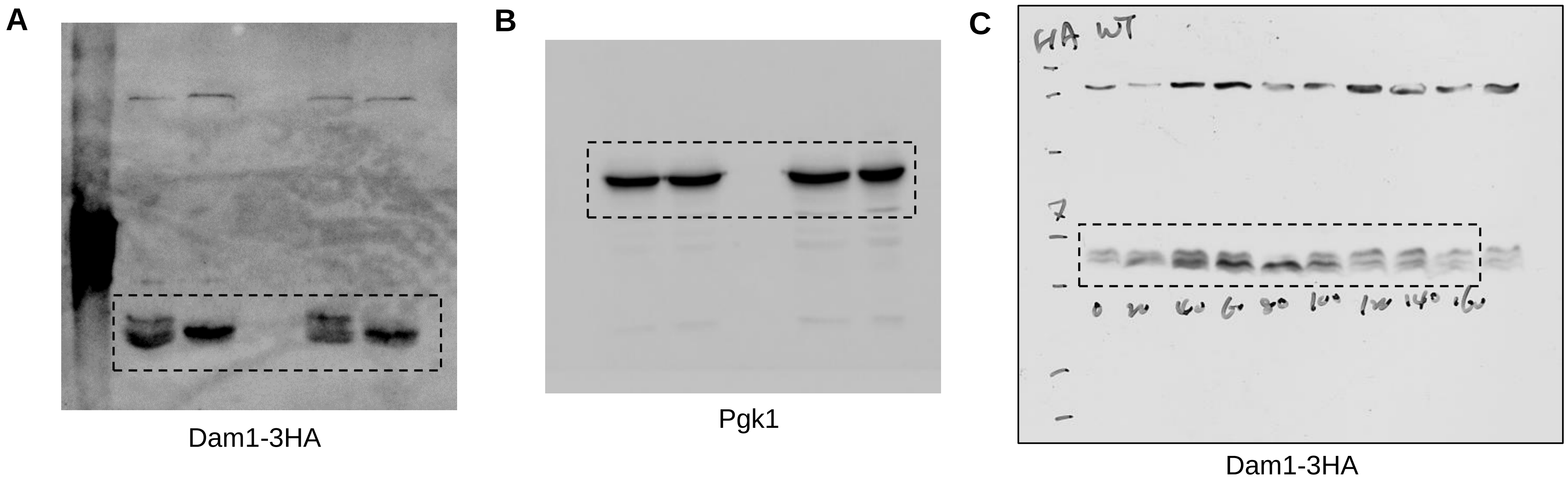

Figure S1: Full-length blots corresponding to Figure 1.
(A) The full-length western blot with anti-HA antibody corresponding to Figure 1A. A non-specific band of high molecular weight is seen in this blot. The cropped area is shown. **(B)** The full-length western blot with anti-Pgk1 antibody corresponding to Figure 1A. The cropped area is shown. **(A)** The full-length blot with anti-HA antibody corresponding to Figure 1B. A non-specific band of high molecular weight is seen in this blot. The cropped area is shown.

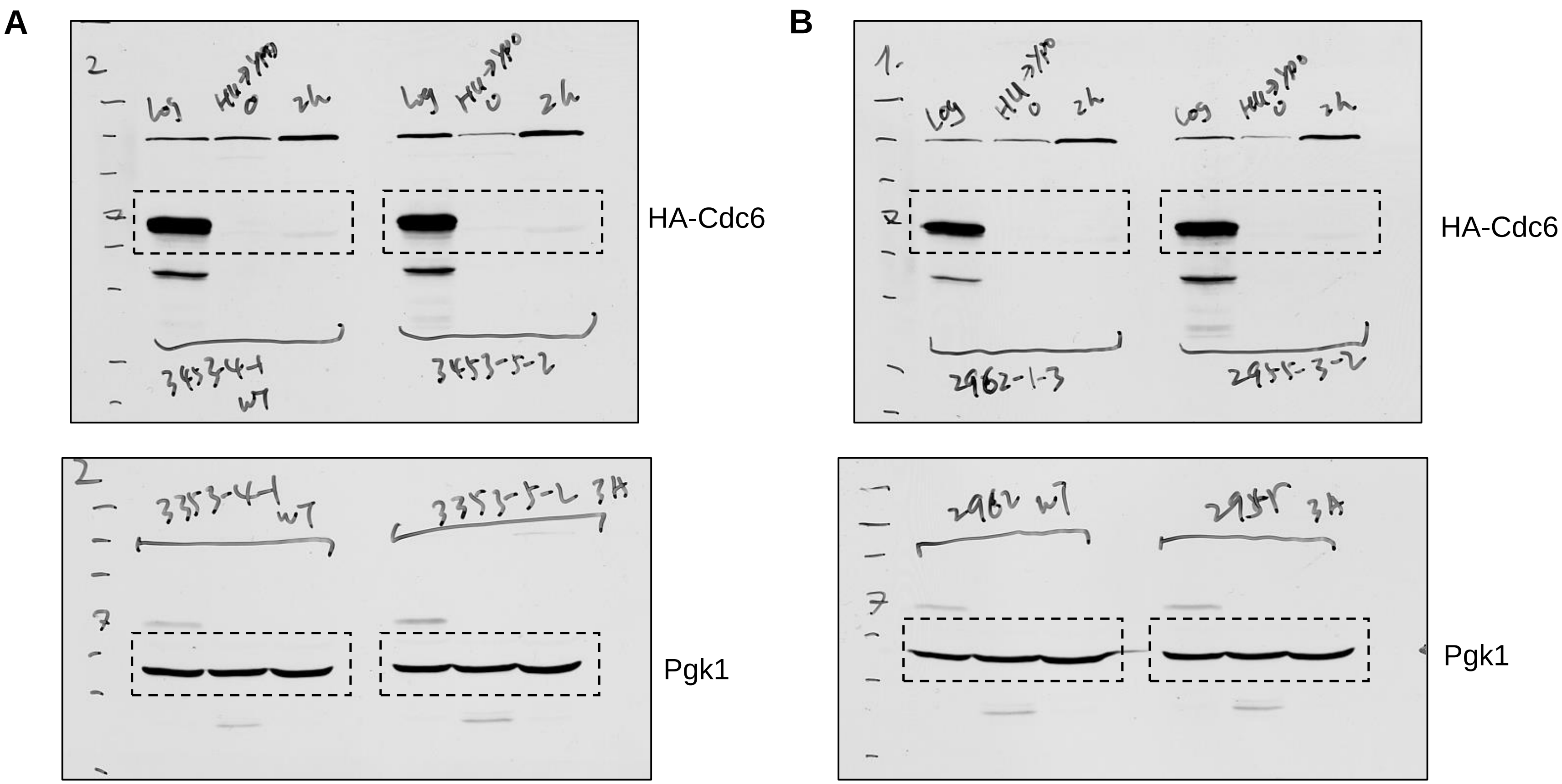

Figure S2: Full-length blots corresponding to Figure 2.
(A) The full-length western blots corresponding to Figure 2C. The western blot with anti-HA antibody indicates HA-Cdc6 protein levels (top). A non-specific band of high molecular weight is seen in this blot. The bottom panel is the full-length blot with anti-Pgk1 antibody. The cropped areas are shown.
(B) The full-length western blots corresponding to Figure 2C. Top panel: western blot with anti-HA antibody. Bottom panel: western blot with anti-Pgk1 antibody. The cropped areas are shown.
